# Phylogenomics of plant genomes: a methodology for genome-wide searches for orthologs in plants

**DOI:** 10.1186/1471-2164-9-183

**Published:** 2008-04-21

**Authors:** Matthieu G Conte, Sylvain Gaillard, Gaetan Droc, Christophe Perin

**Affiliations:** 1 CIRAD, UMR 1096 TA40/03k, Avenue Agropolis, 34398 Montpellier, Cedex 5, France

## Abstract

**Background:**

Gene ortholog identification is now a major objective for mining the increasing amount of sequence data generated by complete or partial genome sequencing projects. Comparative and functional genomics urgently need a method for ortholog detection to reduce gene function inference and to aid in the identification of conserved or divergent genetic pathways between several species. As gene functions change during evolution, reconstructing the evolutionary history of genes should be a more accurate way to differentiate orthologs from paralogs. Phylogenomics takes into account phylogenetic information from high-throughput genome annotation and is the most straightforward way to infer orthologs. However, procedures for automatic detection of orthologs are still scarce and suffer from several limitations.

**Results:**

We developed a procedure for ortholog prediction between *Oryza sativa *and *Arabidopsis thaliana*. Firstly, we established an efficient method to cluster *A. thaliana *and *O. sativa *full proteomes into gene families. Then, we developed an optimized phylogenomics pipeline for ortholog inference. We validated the full procedure using test sets of orthologs and paralogs to demonstrate that our method outperforms pairwise methods for ortholog predictions.

**Conclusion:**

Our procedure achieved a high level of accuracy in predicting ortholog and paralog relationships. Phylogenomic predictions for all validated gene families in both species were easily achieved and we can conclude that our methodology outperforms similarly based methods.

## Background

The availability of complete plant genomes for comparative analysis provides new perspectives in plant development and evolution which will allow an understanding of major trends in plant evolution and species-specific adaptations. The evolutionary history of gene families can be reconstructed and their importance in some morphological innovations evaluated. Sequencing plant genomes along the evolutionary path will be very helpful in elucidating how modifications in expression of key developmental genes and/or coding sequences during evolution are somehow responsible for major trait changes or innovations. Up to now, the genomes of three dicotyledons (*A. thaliana *[1], *P. trichocharpa *[2] and *Vitis vinifera *[3]), one monocotyledon (*O. sativa*) [4] and a moss (*Physcometrilla patens *[5]) have been fully sequenced. The comparison of their gene repertories will help to formulate hypotheses either on conservation or divergence for biological process among several species [6]. Moreover, comparative analysis will be of crucial importance for all species of agronomical interest in which functional analysis of genes can be tedious. Annotation transfer from model species will be the only way to assign a function to the majority of genes in these species.

Two genes are considered to be orthologs if they are separated by a speciation event. Although this definition has nothing to do with biological function, it is commonly assumed that orthologs have equivalent functions in different species. Paralogs are homologs separated by duplication and may have biologically different functions. Co-orthologs/Ultraparalogs are two or more genes in one species that are together orthologous to one or more genes in another species (see [7] for a general discussion of these definitions). Co-orthologs/Ultraparalogs have a strong probability of sharing a very similar function in both organisms. They are often related to phenomena of genetic redundancy, especially when they result from intra-species duplication by segmental and/or tandem duplication. However, gene family evolution is not limited to duplication; it can also include gene loss and rearrangement. This complicates inference of orthologous and paralogous relationships [8] and can even obscure the definition of boundaries between species for prokaryotes.

Identifying orthologs and distinguishing them from paralogs is the key problem. How can we identify the set of orthologs between two or more species? The method follows a two-step process. Firstly, protein sequences from at least two organisms have to be grouped into evolutionarily-related gene families, since the majority of plant protein are encoded by members of multigene families. A gene family is a cluster containing the complete set of homologs and only homologs. The problem of accurate clustering is complex. Most plant gene family databases published so far are partial and contain only specific families (see for instance [9-11]). This clustering step is a critical issue as misassignment of weakly or non-homologous sequences will generate a poorly resolved tree as these sequences are not evolutionarily related. When an accurate catalogue of gene families across species is available, the second step is to predict orthologous relationships between family members both within a given family and across species. Two methods are commonly used, one based on similarity methods and another using phylogenetic analysis. Pairwise similarity comparisons are commonly achieved using BLAST [12] and have been used to develop several ortholog databases [13-15]. For multigene families with recent duplication events, similarity methods fail to distinguish paralogs from orthologs. On the other hand, phylogenetic analysis of homologs is the most straightforward way to identify orthologs. This strategy, applied to full genomes, was named phylogenomics by Dr. Eisen [16,17]. Several powerful tools have recently been developed specifically for phylogenomics, including Resampled Inference of Orthologs (RIO) [18]. RIO uses a procedure known as "tree reconciliation" and compares the gene tree to the species tree topology. A minimal duplication or speciation parsimony principle is then applied to reconcile both trees [18]. Even if they have greatly helped to automatically analyze full genomes, all the phylogenetic methods available are less efficient than similarity based methods [19,20], suggesting a complex set of pitfalls in the phylogenetics approach that needs to be overcome. A full methodology that can be applied on raw data, i.e. using gene sequences of full plant genomes, is still missing to our knowledge.

Thus, all methods for orthology prediction, pairwise comparison and phylogenomics suffer from important drawbacks and need to be improved. We developed a full genome-wide phylogenomics procedure for plant ortholog predictions including:

(i) An efficient methodology for gene family clustering of complete genomes with semi-automatic curation.

(ii) A generic and optimized phylogenetic pipeline for ortholog inference

(iii) A validation method using a test set of orthologs and paralogs to demonstrate that our strategy outperforms ortholog predictions of pairwise methods.

This procedure was successfully applied to predict orthologs between complete *Arabidopsis thaliana *and *Oryza sativa *genomes and will be a guideline for future complete plant genomes ortholog predictions. Future extensions of this procedure to integrate new plant genomes are also discussed.

## Results

### Genome-wide search for plant homolog gene families

#### Genome-wide clustering of homologs in gene families

We first evaluated GCD [21] and PlantTribes [22] as gene family databases for phylogenomic inference. These two plant gene family databases were constructed recently using an automatic clustering approach. The GCD database uses two complementary approaches to produce *A. thaliana/O. sativa *clusters. The first applies BLASTCLUST [12] while the second uses a PFAM domain combination [23]. PlantTribes presents *A. thaliana*/*O. sativa*/*P. Trichocarpa *gene clusters developed with the TribeMCL software [24]. This method relies on the Markov cluster (MCL) algorithm for the assignment of proteins to families based on pre-computed sequence similarity information. Transcription factor (TF) clusters from GCD and PlantTribes were compared to 45 TF gene families of DATF/DRTF [9,10]. DATF and DRTF are manually curated plant transcription factor databases for *A. thaliana *[10] and *Oryza sativa *[9] respectively. A simple parameter was defined to compare completeness and presence of non-homologous sequences in GCD and PlantTribes clusters. Firstly, we identified the GCD and PlantTribes clusters containing the largest number of sequences for each TF family. A cluster was declared incomplete when the number of sequences it contained was below 50% of that in corresponding DATF/DRTF gene family. Similarly, a cluster was declared to contain non-homologous sequences when additional gene members represented more than 50% of the original size of the gene family in DATF/DRTF [9,10].

Different TF gene families were subdivided into several clusters in GCD and PlantTribes. Even with the lowest BlastClust identity parameter (35%) the cluster efficiency of BlastClust was rather poor (Table 1). We found nine incomplete families suggesting that most of the gene family members were separated into several small clusters. The PFAM strategy was more efficient as only two families were subdivided compared to DATF/DRTF. Several clusters contain non-homologous sequences in GCD and PlantTribes. Six clusters, all containing multi-domain proteins, contained a large proportion of non-homologous sequences in GCD (PFAM clustering strategy). As this strategy groups sequences with a similar PFAM combination, this can lead to incorrect clustering when a domain does not define itself as a gene family or when there is no protein domain identified for a given gene family. In PlantTribes, four families (Table 1) also contained non-homologous sequences. Thus, we estimated that GCD and PlantTribes could not be used directly for phylogenomic inference. TribesMCL performs better than the BlastClust and PFAM approach, but there are still some clusters subdivided in PlantTribes compared to DATF/DRTF.

**Table 1:** Clustering of 46 TF in PlantTribes (PT) and Genome Clustering database (GCD) compared to DAFT and DRTF databases.

		***Arabidopsis thaliana***				***Oryza sativa***			
			
**FT NAME**	**IPR**	**DAFT**	**PT**	**GCD**		**DRTF**	**PT**	**GCD**	
							
				**BC**	**PF**			**BC**	**PF**
**EIL**	IPR006957	6	6	6	6	9	10	10	12
**BBR/BPC**	IPR010409	7	8	10	12	4	4	5	5
**S1Fa-like**	IPR006779	3	3b(3)	3	3	2	2	5	5
**BES1**	IPR008540	8	6	9	10	6	5	4	5
**GIF**	IPR007726	3	3	2	3	3	3	2	3
**TCP**	IPR005333	23	23b(2)	27b(15)	27	21	21b(2)	25b(14)	26
**PLATZ**	IPR006734	10	9	9	10	16	16	17	19
**GeBP**	IPR007592	21	18	21b(8)	21	15	21b(4)	15b(10)	16
**SRS**	IPR007818	10	10	11	12	5	5	5	5
**Whirly (PBF-2-like)**	IPR013742	2	3	3	1	1	2	3	0
**AUX/IAA family**	IPR003311	29	28	33	34	31	33	41	41
**CCAAT-HAP3**	IPR003957	11	12	17	37a	12	12	12	29a
**CCAAT-HAP2**	IPR001289	10	10	18	19	11	9	17	19a
**C2C2-DOF**	IPR003851	36	36	40b(16)	40	30	29	34b(18)	33
**C2C2-YABBY**	IPR006780	5	5	6	6	8	7	6	8
**TAZ**	IPR000197	9	11	5	5	6	8	3	4
**TUB/TLP**	IPR000007	11	11	11	10	14	15	16	14
**SBP**	IPR004333	16	17	26b(10)	24	20	18	20b(13)	21
**HSF**	IPR000232	23	23	22	25	29	29	37	39
**AP2/EREBP**	IPR001471	146	110	113	150	165	108	108	170
**NAC**	IPR003441	107	90	92	119	131	92	85	152
**bZIP**	IPR004827	72	185b(12)	82b(22)	84	84	205b(19)	64b(22)	90
**MADS**	IPR002100	104	71	69	65	64	65	62	44
**WRKY**	IPR003657	72	71	105b(30)	81	98	99	96b(37)	104
**GRAS**	IPR005202	33	32	25	34	55	63	39	57
**AS2/LOB**	IPR004883	42	36	35	45	36	29	21	37
**MBF1**	IPR001387	3	3	3	3	2	2	2	2
**Nin-like**	IPR003035	14	61a	9	10	13	315a	13b(9)	13b(2)
**ZF-HD**	IPR006456	16	16	13	17	15	15	8	15
**CPP**	IPR005172	8	10	3	9	11	18	3	11
**ARF**	IPR011525	22	25	24	19	26	29	26	22
**ZIM**	IPR010399	18	10	26b(9)	20	18	13	10	19
**VOZ**	IPR009105	2	2	2	1	2	2	2	0
**ULT**	IPR000770	2	2	2	1	2	2	2	0
**GARP-G2-like**	IPR001005 IPR009057	43	73	22	260a	46	80	56b(23)	244a
**GARP-ARR-B**	IPR001789 IPR009057	10	73a	8	14	8	80a	4	9
**LUG**	IPR006594 IPR011046	2	76a	5	227a	6	74a	3	207
**CAMTA**	IPR000048 IPR002110 IPR005559	6	6	4	3	6	8	5	5
**ALFIN**	IPR001965 IPR011011	7	7	7	46a	10	10	10	56
**CCAAT-DR1**	IPR003958 IPR009072	2	12a	17a	37a	1	12a	12a	29a
**CCAAT-HAP5**	IPR003958 IPR009072	13	10	15	37a	16	12	17b(8)	29a
**NZZ**		1	1	1	1	1	1	0	0
**GRF**		9	9	9b(7)	9b(8)	12	16	12b(8)	12b(11)
**HRT-like**		2	3	3b(2)	2b(2)	1	1	1	1
**MYB3R and R2R3**	IPR001005 IPR009057	150	222	118	260	129	221	86	244

a: Cluster containing more than 50% of sequences in comparison with the total sequence family members present in DATF/DRTF; b: Total number of sequences identified in several subgroups (number of subgroups in brackets) for the DATF/DRTF corresponding gene list; PF by PFAM domains; BC by BLASTCLUST. Note that for all groups, the sequence number differences found with DATF/DRTF are mainly due to data source versions used.

The incompleteness of TF in PlantTribes can be corrected using relaxed TribesMCL parameters. Two major parameters are used for gene clustering in TribesMCL: the classical BLASTALL E-value and the Inflation value. The Inflation value is an indirect indication of "tightness" of clustering, such that the higher the inflation, the more stringent the clustering [24,25]. Several combinations of BLASTALL E and Inflation values were tested. We evaluated the completeness of the automatic clustering procedure by comparing the TF families described in DATF and DRTF with clusters automatically generated by the TribeMCL procedure with the relaxed parameters (BLASTALL = 10, I = 1.2). In the DATF and DRTF databases, 63 families of TFs are described. The automatic clustering with relaxed parameters procedure generated 45 families (71%) in full concordance with DATF and DRTF databases (Additional File 1) and all of the families identified as badly clustered before (Table 1) in GCD and PlantTribes were correctly assigned.

#### Manual curation and mapping of At/Os clusters to plant gene families

**Figure 1:**
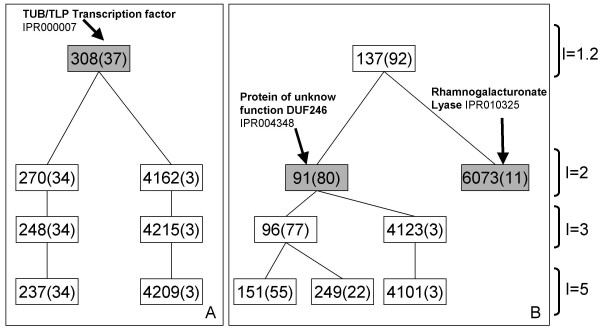
**Examples of cluster curation generated automatically by TribeMCL**. (A) An example of a gene family identified for the cluster 308 at Inflation = 1.2 corresponding to the TUB/TLP Transcription factor gene family. (B) Another example where two consistent gene families were identified for the Clusters 91 and 6073 at higher Inflation (I = 2). Cluster 137 (I = 1.2) groups members from these two families and thus cannot be considered as a gene family. Inside the box is the cluster id and in brackets is the number of At/Os sequences inside the cluster.

Clusters generated by TribesMCL were manually mapped to gene families using external evidence including various databases (TAIR, DATF/DRTF, INTERPRO families, KEGG) and in a few cases publications describing plant gene families. Curation of the automatically generated clusters was then always necessary; for instance in TF families, 14% of the clusters in one-to-one correspondence with DATF/DRTF were identified at inflation values greater than 1.2 (Additional File 1). Gene family assignment of several thousand clusters was done easily using a custom annotation database developed specifically for this purpose [26]. The curation procedure starts by analysing all clusters produced at the inflation value 1.2. For instance, cluster 308 contains all TUB/TLP plant transcription sequences based on the INTERPRO domain IPR000007 and was annotated as the TUB/TLP Transcription factor gene family (Figure 1A), while cluster 137 contains sequences from two separate gene families (Figure 1B) and thus does not represent a unique gene family. All sequences in cluster 137 belong to two sub-clusters at a higher inflation value (I = 2) and which correspond clearly to two gene families: one composed of proteins of unknown function DUF246 (INTERPRO family domain IPR004348) and the Rhamnogalacturonate lyase family (INTERPRO family domain IPR010325) (see Figure 1B). Global family assignment followed a similar process: we first curated all clusters generated at the lowest inflation value (I = 1.2) and if a cluster clearly contained sequences from different families, sub-clusters were curated. The procedure was repeated until we obtained clusters matching gene families.

A total of 21,038 gene clusters were assembled using the TribesMCL pipeline software at four levels of inflation. Up to now, 6,421 clusters have been manually annotated including 64 from DRTF [9] and DATF [10] TF databases, 492 from TAIR [27], 1,903 from the INTERPRO family list [28] and 981 from the KEGG [29] database. The most probable molecular function (or putative molecular function) was tentatively attributed to each gene family. During the manual curation, we also identified species-specific clusters (either *O. sativa *or *A. thaliana*). We considered that a cluster was species-specific if at least two sequences belonging to a single species were grouped together at the lowest inflation value (I = 1.2). As such, we found 703 *O. sativa *and 116 *Arabidopsis thaliana *specific clusters. In several cases, these families were already defined as species-specific based on the family's INTERPRO domain. For example, the ASR family is missing in *A. thaliana *and we identified an *O. sativa *specific cluster matching the IPR family domain IPR003496. Similarly, the plant self-incompatibility S1 family was identified as an *A. thaliana *specific cluster with 40 members (IPR family domain IPR010264). Automatic clustering was incorrect in only 6% of the cases (392 clusters among the 6421 manually annotated clusters) and we had to reconstruct these clusters manually. We then redesigned the clustering of these families by creating and/or destroying several clusters. Semi-automatic production of gene families was easily achieved in a short time, creating the largest existing catalogue of plant gene families (6421). All clusters from *A. thaliana *and *Oryza sativa *containing more than three sequences and generated at lower inflation values (I = 1.2) were annotated. The full catalogue is accessible at GreenPhylDB database [26].

### Genome-wide search for plant orthologs

Our goal was to design an optimal phylogenomics pipeline to predict orthologs not only between *A. thaliana *and *O. sativa*, but also with other plant species. We had to develop model alignments as we started from gene families and not from a source of curated alignment as in PFAM [23]. One of the critical problems was to automatically eliminate and filter misannotated sequences in a given gene family to build an optimal model alignment. This filtering step eliminated alternatively-spliced products and misannotated or too highly divergent sequences. The alignment procedure should allow alignment of conserved regions separated by large gaps, but also of relatively divergent sequences as most of the curated gene families are multi-domain containing proteins. A specific masking step had to be included in order to optimize the alignment for phylogenetic construction by removing poorly informative amino-acid positions.

#### Greenphyl phylogenomics pipeline

**Figure 2:**
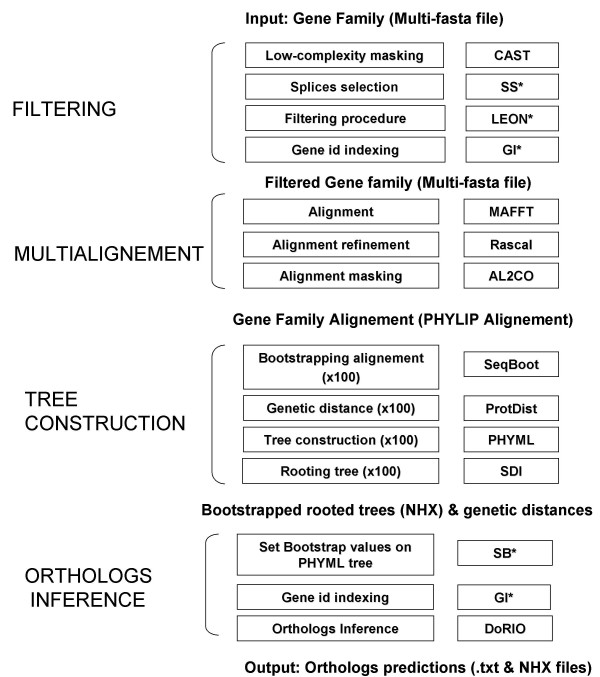
**The phylogenomic pipeline GreenPhyl**. Left: the major steps of the phylogenomics pipeline. Right: the software integrated in GreenPhyl. *, custom software. SS, splice selection, GI, gene id indexing. SB, set bootstrap values.

GreenPhyl is a pipeline written in Perl using 15 different types of software, including several custom C software programs (Figure 2). GreenPhyl input is a multi-fasta file with gene identifiers tagged with a species code. As output, GreenPhyl generates all the ortholog predictions for each sequence contained in the original multi-fasta file. Compared to already published phylogenomic pipelines, three steps were substantially modified.

Misclassified or incomplete sequences have significant impact on phylogenomic constructions. These divergent sequences are interpreted as sequences that evolve more rapidly compared to other family members. This will cause Long Branch Attraction (LBA) effects during tree construction, either due to unequal rates of evolution or annotation errors. Thus, they have to be automatically identified and rejected for each family. We added an automatic filtering step combining the Muscle [30] and LEON [31] softwares. Briefly, in a given cluster, each sequence is successively fixed as a "family model sequence" and tested against all other family members using LEON. This method, which gives as output a list of "non-homologous sequences," has been applied to each analysed family. We considered as non-homologous a gene rejected by more than 96% of other family members. This filtering step rejected on average 60% (computed on 56 TF families) of misannoted TF sequences, based on the absence of the typical TF INTERPO domain. For example, this method rejects 100% of non-homologous sequences in the GRAS family (8 sequences) and 85% in the MADS family (25 sequences). Sequences that shared a degenerate domain or did not contain the typical domain were easily detected by the LEON filtering system as they could not be aligned correctly with other family members. This filtering system also deals with multi-domain family problems. In the MADS family for instance, the K-box domain (IPR002487) is always associated with the MADS domain. The LEON filtering system rejected the 10 sequences that only present the K-box domain without the MADS domain. Finally, for each locus, we kept only the splicing form with the best similarity score with all other family members as estimated by the Checksum value of the multi-sequences Format (MSF).

Multi-alignment is one of the major steps in phylogenomic construction. The objective is to identify and align the characteristic domains of the gene families. Many alignment algorithms exist. Our objective was to select a program able to align multi-domain families. We selected MAFFT [32-34] with the E-INS-i option that can align conserved regions separated by large gaps or very divergent sequences, a feature needed for multi-domain alignment [35]. This method has been successfully tested (CPU time and accuracy) by computer simulations and the BAliBASE [36] benchmark tests in comparison with several existing methods. The RASCAL program [37] was used to optimize the multiple alignment produced by MAFFT. This improvement was also quantified on a BAliBASE test set. Moreover, MAFFT offers a range of multiple alignment methods including alignment of a very large number of sequences, a feature needed either for very large multigene families or when a large number of species is employed. Finally, we applied a masking procedure to the optimized alignment to detect and remove amino acid columns/positions containing either no or a low phylogenetic signal. Our workflow uses a modified version of the AL2CO software for calculation of positional conservation [38]. The amino acid positions retained for phylogenetic constructions share a minimum conservation index of 2 together with a percentage of gaps below 50%. The multi-alignment process was designed to run with any kind of gene family structure, from single domain to multi-domain containing proteins. We additionally generated a full-length HMM profile from the RASCAL optimised alignment with the HMMER package [39] to provide a simple way to identify new members of the gene family in the future using a library of HMM profiles.

PHYML [40] is one of the fastest maximum-likelihood tree reconstruction methods for generation of large trees with an acceptable CPU computing time. PHYML first constructs a BioNJ tree using the Neighbor-Joining tree algorithm and then optimizes this tree to improve its likelihood by successive iteration. The SDI unrooted method [41] from the RIO package [18] was used to root the generated gene trees. Finally, we looped DoRIO [18] on bootstrapped rooted trees for ortholog inference for each sequence. We also developed a new plant species tree, based on a RIO [18] published tree and including the top 100 plant species based on NBCI rankings using the number of stored sequences in NCBI (see Additional files 2 &3).

#### Validation of the phylogenomics procedure using an ortholog test set and comparison with pairwise methods

Even if the phylogenomics approach was theoretically the most powerful methodology to predict orthologs, up to now the most efficient approaches have been pairwise methods [19,20]. In order to demonstrate that our phylogenomics methodology outperforms pairwise methods, we compared ortholog predictions against a test set of manually curated orthologs.

We first reviewed the literature to find ortholog evidence between *Oryza sativa *and *Arabidopsis thaliana *(true positives). We then extracted and compared ortholog predictions for these genes from three methods, BBMH (Best Blast Mutual Hits) and Inparanoid [42] which are methods based on pairwise comparisons and GreenPhyl (Table 2). A total of 35 true positive orthologous relationships (according to our counting procedure: See *Materials and Methods*) were selected from bibliographic references, including 11 one-to-one, six one-to- two and two two-to-three relationships. GreenPhyl predicted 37 orthologous relationships and missed three, Inparanoid predicted 23 and missed 14, while BBMH predicted only 17 orthologous relationships and missed 18. Thus, our phylogenomics pipeline outperformed Inparanoid and BBMH methods, especially for several-to-several orthologous relationships for which the similarity methods often miss some or all ultraparalogs. Ultraparalog definitions from Zmasek are as follows [18]: "Given a rooted gene tree with duplication or speciation assigned to each of its internal nodes, two sequences are ultra-paralogous if and only if the smallest subtree containing them both contains only internal nodes representing duplications" (see also materials and methods for Zmasek definitions).

**Table 2:** True positive ortholog test set and ortholog predictions of GreenPhyl (GP), Inparanoid (INP) and BBMH (BH).

***Arabidopsis thaliana***		***Oryza sativa***		**Orthologs**		**True positive**			**Missing**		
				
**TAIR id**	**Alias**	**TIGR id**	**Alias**	**n/n**	**PMID**	**GP**	**BH**	**INP**	**GP**	**BH**	**INP**
At5g20240.1	PI	Os05g34940.1	OsMADS4	1/2	14704206	1/2	1/1	1/2	0	1	0
		Os01g66030.1	OsMADS2								
At3g54340.1	AP3/DEF	Os06g49840.1	SPW1	1/1	12506001	1/1	1/1	1/1	0	0	0
At1g24260.1	SEP3	Os09g32948.1	OsMADS8	3/2	16968881; 17205197; 10821278	3/2	1/1	1/1	0	5	5
At3g02310.1	SEP2	Os08g41950.2	OsMADS7								
At5g15800.1	SEP1										
At4g18960.1	AG	Os01g10504.1	OsMADS3	1/2	16326928	0/3	0	1/1	2	2	1
		Os05g11414.3	OsMADS58								
At2g45660.1	SOC1	Os03g03070.1	OsMADS50	1/1	15144377	0/3	0	0	1	1	1
At1g14920.1	GAI	Os03g49990.1	SLR1	2/1	11340177; 11826293	3/1	1/1	1/1	0	1	1
At2g01570.1	RGA1										
At1g55580.1	LAS	Os06g40780.1	MOC1	1/1	12687001; 12730136	1/2	1/1	1/1	0	0	0
		Os02g10360.1									
At3g54220.1	SCR	Os12g02870.1	OsSCR	1/2	12974810	1/2	1/1	1/2	0	1	0
		Os11g03110.1									
At4g37650.1	SHR	Os03g31880.1	OsSHR	1/2	12974810	1/2	1/1	1/1	0	1	1
		Os07g39820.1									
At3g11260.1	WOX5	Os01g63510.1	QHB	2/1	12904206; 14711878	2/1	1/1	1/1	0	1	1
At5g05770.1	WOX7										
At4g16280.3	FCA	Os09g03610.2		1/1	16240176	1/1	1/1	1/1	0	0	0
At4g00650.1	FRI	Os03g63440.1		1/1	12667866	1/1	1/1	1/1	0	0	0
At2g44990.1	MAX3	Os04g46470.1	HTD1	1/1	17092317	1/1	1/1	1/1	0	0	0
At2g42620.1	MAX2	Os06g06050.1	OsMAX2	1/1	15659436	1/1	1/1	1/1	0	0	0
At5g03280.1	EIN2	Os07g06130.1	OsEIN2	1/1	15047876	1/2	1/1	1/3	0	0	0
		Os03g49400.1									
At5g47120.1	Bi1	Os02g03280.1	OsBi1	1/1	10618494	1/1	1/1	1/1	0	0	0
At2g27550.1	CEN	Os11g05470.1	RCN1	2/3	8974397; 12148532	2/4	1/1	1/2	0	5	4
At5g03840.1	TFL1	Os04g33570.1									
		Os02g32950.1	RCN2								
		Os12g05590.1	RCN3								
At1g22770.1	GI	Os01g08700.1	OsGI	1/1	12700762	1/1	1/1	1/1	0	0	0
At5g61380.1	TOC1	Os02g40510.1	PRR1	1/1	14634161	1/1	1/1	1/1	0	0	0

TAIR, TIGR id = accession number from TAIR and TIGR; orth = ortholog relations identified in the literature. GP = Greenphyl; BH = BBMH; INP = Inparanoid; true positives = ortholog relation; missing = ortholog missed by GP, BH or INP. PMID = Pubmed id

We also identified 'true negative' orthologous relationships from the literature. These represent a set of genes known to be species-specific with no orthologous relationships to the other species (Table 3). In this particular case, GreenPhyl easily identifies ultraparalog relationships when no orthologs are detected. For example, GreenPhyl predicted that LHS1 is a rice-specific gene associated with 2 additional ultraparalogs (Table 3). In *Arabidopsis thaliana*, the flowering locus (FLC) has no ortholog in rice, but several closely related *Arabidopsis thaliana *ultraparalogs were identified (MAF-like for MADS affecting flowering) and are known to be involved together with FLC in the flowering pathway [43]. BBMH and Inparanoid incorrectly predicted an orthologous relationship for the *Arabidopsis thaliana *gene VRN1 (Table 3). These results suggest that absence of prediction by GreenPhyl is correct and this is a true negative. Moreover, GreenPhyl predicted 15 additional rice sequences as ultraparalogs.

**Table 3:** True negative ortholog test set and ortholog predictions of GreenPhyl (GP), Inparanoid (INP) and BBMH (BH).

***Arabidopsis thaliana***		***Oryza sativa***		**Literature**		**UP prediction**		
			
**TAIR id**	**Alias**	**TIGR id**	**Alias**	**UP**	**PMID**	**GP**	**BBMH**	**INP**
-	-	Os03g11614.1	LHS1	0/3	16099195; 10852934	0/3	0	0
-	-	Os06g06750.1	MADS5					
-	-	Os03g54170.1	MADS34					
At5g10140.1	FLC	-	-	6/0	12667866;12724541; 15695584	6/0	0	0
At5g65060.2	MAF3	-	-					
At5g65070.1	MAF4	-	-					
At5g65050.1	MAF2	-	-					
At1g77080.3	MAF1	-	-					
At5g65080.1	MAF5	-	-					
At3g18990.1	VRN1	-	-	2/0	12667866;16549797	2/0	1/1	1/2
At1g49480.1	RTV1	-	-					
At4g16845.1	VRN2	-	-	1/0	12667866	1/0	0	0

UP = Ultraparalogs identified in the literature; PMID = Pubmed id; GP = GreenPhyl; INP = Inparanoid

**Figure 3:**
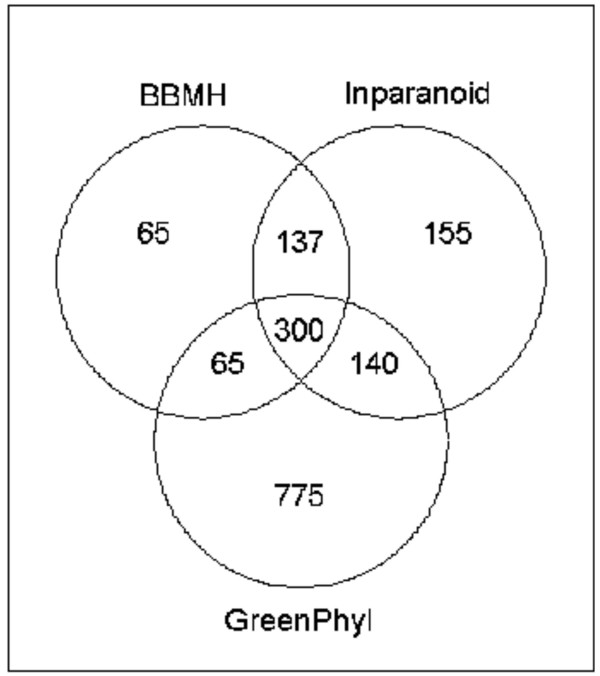
Venn diagram of ortholog prediction between GreenPhyl, BBMH and Inparanoid at a threshold of 50%.

Thus, our phylogenomics pipeline achieves the highest number of ortholog predictions with good overlap of predictions by similarity methods. We ran the GreenPhyl pipeline on 56 TF families (3314 genes) validated by our clustering procedure and compared the results with Inparanoid and BBMH predictions (Figure 3, and see additional file 4). These analyses predicted a total of 1637 non-redundant ortholog relationships: 567 by BBMH, 732 by Inparanoid and 1280 by GreenPhyl. Since BBMH only finds one-to-one (1/1) relationships, ortholog prediction using this method is obviously underestimated. Inparanoid slightly outperforms BBMH by 22% while GreenPhyl predicts approximately twice as many orthologs when compared with any other similarity method. In total, 77% of the 1637 ortholog predictions are detected by GreenPhyl, 45% by Inparanoid and 35% by BBMH. Moreover, 65% and 60% of the BBMH and Inparanoid predictions are achieved by GreenPhyl, while all similarity methods together can achieve only 40% of the GreenPhyl predictions. GreenPhyl-specific predictions represent the highest proportion of the non-redundant set (775, 47%) followed by Inparanoid (155, 9.4%) and BBMH (65, 4%).

As GreenPhyl detected a greater number of orthologous relationships, we asked why it is more efficient than Inparanoid and whether these additional orthologs are *bona fide *orthologs. Firstly, as the threshold applied for Inparanoid and GreenPhyl was arbitrary fixed at 50%, we asked whether some of the orthologs specifically detected by GreenPhyl or Inparanoid were *in fine *detected by the other methods at slightly lower threshold values. A total of 18% (141) of the supplementary orthologs, detected originally by GreenPhyl, were identified by Inparanoid using a 30% threshold. Similarly, 30% (87) of the orthologs detected by Inparanoid are recovered by GreenPhyl using a 30% threshold.

**Figure 4:**
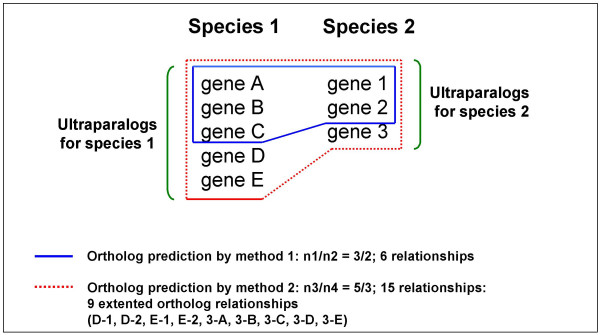
Extended orthologs relationships.

Nevertheless, GreenPhyl detects 649 orthologous relationships never found by any similarity based method even at lower thresholds. These GreenPhyl-specific predictions could have two origins. They could be due to a more efficient detection of complex orthologous relationships corresponding to intra-specific duplications (Figure 4) with multiple co-orthologs. Alternatively, these orthologs could be totally new predictions. In order to obtain better insight on specific ortholog prediction by GreenPhyl, we classified these predictions in two classes:

(i) If one method predicts an n1/n2 orthologous relationship and the other method predicts an n3/n4 relationship, then "extended orthologs" are n1/n2 relationships not included in n3/n4 ones (see Figure 4).

(ii) If one method predicts an n1/n2 orthologous relationship never predicted by the other method, then the new orthologs are considered as "in-specific orthologs"

We evaluated the number of 'extended' and 'in-specific' orthologs for Inparanoid and GreenPhyl using the list of 56 TF families previously used (additional file 5). A total of 680 orthologs (486 extended orthologs and 194 in-specific orthologs) predicted by GreenPhyl are missing in Inparanoid while 171 (4 extended orthologs and 167 in-specific orthologs) predicted by Inparanoid are missing in GreenPhyl. Strikingly, 2% (4) of the Inparanoid orthologs missed by GreenPhyl are "extended orthologs" while 71% (486) of the GreenPhyl orthologs missed by Inparanoid are "extended orthologs". So GreenPhyl seems to deal better with more complex relationships and is able to identify co-ortholog relationships for each species.

Nevertheless, we decided to evaluate if these additional co-orthologs correspond to true co-ortholog predictions. We used an indirect validation method. Segmental and tandem duplication information was downloaded from TIGR and OryGenesDB, respectively (additional file 6) and we evaluated how many of these intra-species duplications are detected as ultraparalogs and in-paralogs by GreenPhyl and Inparanoid respectively. If most of the co-orthologs detected specifically by GreenPhyl really correspond to intra-species duplications, we expect to identify most of the tandem and segmental duplications as GreenPhyl Ultraparalog predictions. A total of 2228 ultraparalogs were recovered by GreenPhyl for the 56 TF families (additional file 6). By data mining of TIGR and OryGenesDB datasets, we identified 705 and 211 segmental and tandem duplicated genes in these 56 TF families. Approximately 70% of the segmental and 78% of the tandemly-duplicated genes were predicted to be ultraparalogs by GreenPhyl as expected while Inparanoid detects only 7.5% of the segmental and 5.7% of the tandem duplications. These results suggest that GreenPhyl is more efficient than Inparanoid in identifying co-ortholog relationships. Moreover, these results strongly suggest that most of the specific orthologs predicted by GreenPhyl are true co-orthologs resulting from intra-species duplications, most of which are missed by Inparanoid. These results indirectly validate most of the specific co-orthologs predicted by GreenPhyl and missed by Inparanoid.

## Discussion

We have successfully developed a complete procedure for ortholog prediction between complete *Arabidopsis thaliana *and *Oryza sativa *genomes. Our strategy includes a simple and fast semi-automatic clustering step and avoids most of the common pitfalls of other clustering procedures. Several others clustering procedures have been described. For instance, RIO [18] starts from PFAM [23] as a source of curated alignments. This strategy has several drawbacks. Firstly, PFAM domains do not always define gene families, as several domains are in fact structural or repeat domains (for instance Leucine Rich Repeat, LRR domain PF00560). Moreover, some gene family members, even for fully sequenced genomes, are not systematically integrated in PFAM. These missing members increase the probability of generating pseudo-ortholog predictions as they are equivalent to species specific gene loss (see below). Moreover, PFAM clusters contain sequences from bacteria, animals and plants. Tree constructions using such a wide range of species can lower the efficiency of ortholog predictions. Based on this, another strategy was to develop a procedure for automatic clustering of full genomes like GCD and PlantTribes. We demonstrated that these methods are not directly suitable for phylogenomic analysis as they contain high proportion of clusters with non-homologous sequences and/or incomplete gene families (see results for GCD [21] and PlantTribes [22] databases). A third solution, used, for instance for the COG database [44], is to create groups of homologs by manual inspection. This procedure is time consuming and is efficient only for already well-defined gene families. Using our simple semi-automatic procedure of clustering, we easily achieved full *A. thaliana*/*O. sativa *cluster curation as gene families suitable for phylogenomics analysis. These gene families are accessible at GreenPhylDB database [26]. Lastly, even when starting with complete genome sequence, our clustering procedure applied in complete genomes can also miss some genes either not yet characterized or annotated. The only way to correct this problem in future is to update the gene family clusters using newly annotated sequences. Our methodology can now be easily extended to other plant sequences and we plan to release a new update of gene family clusters each year as well as the gene family phylogeny in order to correct progressively clustering and orthologs predictions. A simple way is to use the HMM gene family patterns generated on gene family model alignments to assign new plant sequences to curated gene families.

We have developed a phylogenomic pipeline to analyse curated gene families. Some gene models automatically predicted from BAC/PAC sequences are inaccurately predicted, especially in *O. sativa *where the quality of genome annotation is less accurate than in *A. thaliana *[45,46]. For instance, some can still contain transposon elements. Thus, our pipeline was specifically developed to reject these sequences before gene multi-alignment to optimize phylogenetic inference. Phylogenomics procedures, including our procedure, generally do not account for uncertainty in the sequence alignment. Several studies (see for instance [47]), including a recent systematic evaluation of sequence alignment uncertainty for phylogenomics [48], have demonstrated that alignment method has a considerable impact on tree topology. Even if we solved partially this problem by automatically rejecting highly divergent or missannoted sequences and if we choose one of the best alignment software, a set of the alignments generated will be inconsistent. This is neither a problem a data quality nor a problem of alignment analysis but reflects the fact that the phylogenomics analysis does not accommodate with alignment uncertainty [47]. A significant part of the genes will be hard to align and this will result to uncertainty in phylogeny as well as in orthologs inference. There is no simple solution to this problem to date and this is clearly a limitation of the procedure for some of the gene families. A suggested way to solve this problem is to consider alignment as a random variable, a strategy developed into the statistical alignment procedure [49,50].

The phylogenomic pipeline can then be applied directly to a multi-fasta file containing sequences from any gene family. We developed an extended species tree of life, including 100 new plant species, for the tree reconciliation procedure of RIO based on NCBI taxonomy data (see additional data file 1). Recently, several other tree reconciliation software programs have been proposed. These methods do not deal with alignment bootstrapping, but use Bayesian statistics [51] to infer orthologous relations. They share several advantages compared to bootstrapping procedures. They seem to outperform bootstrapping methodology as Bayesian methods also tend to deal with errors in data: a major drawback of all gene tree related methods for ortholog prediction (see for instance [51]). Introduction of bootstrapping alternatives like Bayesian statistics for ortholog inference [51] will be tested and implemented in the next release of the pipeline GreenPhyl as we did not yet evaluated this strategy for genome-wide detection of orthologs. The phylogenomic pipeline is accessible as stand alone software [52]. We also recently release a database, GreenPhylDB containing all the precomputed orthologs predictions and the associated tools [26].

We validated our phylogenomics prediction using three curated test sets and demonstrated that our approach clearly outperforms pairwise methods previously described as the most efficient ortholog prediction tools [19,20]. Gene tree construction is very sensitive to annotation errors and the efficiency of our phylogenomics pipeline is mainly due to the sequences used and the alignment filtering steps. They reject non-homologous or dissimilar sequences before construction of the gene family alignment. Despite these validations on moderate samples, how many ortholog predictions represent 'true' ortholog relations? There is no simple way to evaluate this figure. Indeed, numerous evolutionary events including horizontal gene transfer, gene loss, gene fusion and gene fission can lead to pseudo-ortholog predictions [7]. For instance, differential gene loss in a species can lead to pseudo-ortholog predictions and the only way to infer correct orthologous relationships is to reconstruct the evolution of the gene family including a larger sample of species.

Integrating new plant genomes is then not only needed to correct for the pseudo-ortholog bias described above, but it can also help functional transfer from and to model plant species. Initially, we planned to cluster and run phylogenomic analyses in four plant genomes, but we rapidly observed that annotation quality was a major limiting point. Even for the *O. sativa *genome, we observed numerous misannotated sequences rejected by our filtering system such as transposon elements containing proteins or truncated gene models that greatly hamper clustering and ortholog inference. For instance, we only used the complete genome annotation of the *japonica *cultivar Nipponbare [4], presuming that the indica 93–11 indica WGS genome annotations were less advanced [53] even if a new release has recently been made available [54]. The introduction of newly sequenced genomes, most in the assembly phase, was delayed and we are currently testing two different approaches to integrate new full plant genomes. A first possibility is to produce new model alignments by running the pipeline with *A. thaliana*, *O. sativa *and the sequences coming from other genomes for each gene family. This strategy is time consuming to compute, and producing new fully bootstrapped alignments and matrix genetics distances will rapidly become impossible for large multigene families as new complete plant genomes sequences are added. Introduction of bootstrapping alternatives like Bayesian statistics for ortholog inference [51] will probably become more critical with this strategy. Another simple method involving less computing time is to build an alignment model only for a few plant species, including *A. thaliana *and *O. sativa*, and re-align the new family members. By using precomputed genetic distances, these new sequences can be integrated into the previously computed tree. This strategy was initially introduced in RIO [18] and can probably safely be used to add a few sequences in a model alignment. However, the validity of this approach to integrate sequences for a larger sequence sample needs to be tested, at least for large and small multigenic families. We are currently testing this strategy on a large set of gene families before implementation of this procedure. Nevertheless, currently any user can also decide to run our standalone pipeline version on their set of plant sequences.

## Conclusion

We have developed a simple and fast procedure for ortholog prediction between complete genomes, including semi-automatic gene family clustering and an optimised standalone phylogenomic pipeline. The phylogenomic pipeline can be download at [52] or request from the authors'. The precomputed ortholog predictions and curated gene families for the complete genomes of *A. thaliana *and *O. sativa *are accessible at GreenPhylDB, a database published in a companion paper [26].

## Methods

### Data Sources

#### Plant proteomes

The Institute for Genomic Research (TIGR) pseudo-chromosome reference annotation layers for *Arabidopsis thaliana *(Version 6) and *Oryza sativa *(Version 4) were downloaded from the TAIR [55] and TIGR [56], respectively.

#### Tandem and segmental duplications

The full list of homologous sequences found in segmental duplicated regions were downloaded from the TIGR web site with a maximum length distance permitted between collinear gene pairs of 100 kb for *Oryza sativa *[57] and *Arabidopsis thaliana *[58] respectively. The full list of tandem duplicated genes was extracted from the 'paralog clusters' feature layer of OryGenesDB [59].

### Family clustering

#### Clustering

To begin, all protein sequences from *Oryza sativa *and *Arabidopsis thaliana *genomes were grouped into gene families using TribeMCL [24]. This software uses a Markov cluster (MCL) algorithm for grouping proteins into families based on a pre-computed sequence pairwise similarity matrix. We used several TribeMCL parameters Inflation (1.2, 2, 3 and 5) and BLAST E-values (E = 10) for family clustering. Then, for each sequence, low complexity regions were masked using the CAST software [60]. Finally, InterproScan was used to identify protein domains for all genes [28].

#### Filtering

We used an automatic filter to remove the most divergent sequences inside each cluster. A pre-alignment was generated with the MUSCLE alignment software [30] to select the optimal splice form using the best Checksum value. Then, LEON [31] was used to remove highly divergent sequences from the alignment. Each sequence was successively fixed as a "family model sequence" and LEON produced an output list of "non-homologous sequences." We loop LEON for all the sequences of the family and automatically removed the sequences rejected by more than 96% of other family members.

### Phylogenomic analysis

#### GreenPhyl Pipeline

Figure 2 summarizes the GreenPhyl workflow and the description of softwares included in the pipeline.

#### Alignment

A full alignment with the filtered sequences was produced with MAFFT using the E-INS-i MAFFT option [35]. The RASCAL program [37] optimizes the multi-alignment produced by MAFFT. We applied AL2CO software for calculation of positional conservation to mask the generated optimal alignment [38]. The amino acid columns retained for phylogenetic constructions shared a minimum conservation index of 2 together with a percentage of gaps below 50%.

#### Tree construction

PHYML [40] first constructs a BioNJ tree using the Neighbor-Joining tree algorithm and then optimizes this tree to improve the likelihood at each iteration.

#### Identification of speciation and duplication events

We used the Resampled Inference of Orthologs (RIO) procedure to detect orthologs [18]. RIO is based on a bootstrap resampling method to check robustness of ortholog predictions based on the phylogenetic tree. The SDI unrooted algorithm [41] was first used to predict speciation and duplication events. Orthologs, ultraparalogs and subtree-neighboring predictions for each sequence of the tree were generated using the DoRIO procedure. We used ATV JAVA software [61] to display phylogenetic trees with the bootstrap values calculated by DoRIO. Paralog and ortholog associations were considered to be significant if the supporting bootstrap value is above 50%.

#### Phylogenomics concepts

There is more than just ortholog prediction, and three new concepts were defined as super-orthologs, ultra-paralogs and subtree-neighbors by Dr Zmasek [18].

##### Super-orthologs

"Given a rooted gene tree with duplication or speciation assigned to each of its internal nodes, two sequences are super-orthologous if and only if each internal node on their connecting path represents a speciation event." Super-othologs are orthologs in one-to-one correspondence. Super-orthologs represent then a subset of its orthologs for a given sequence. They have the highest probability of sharing a similar function in several species and can be used with high confidence for a direct annotation transfer.

##### Ultraparalogs

"Given a rooted gene tree with duplication or speciation assigned to each of its internal nodes, two sequences are ultra-paralogous if and only if the smallest subtree containing them both contains only internal nodes representing duplications." Ultraparalogs are mostly paralogs that have undergone recent duplications in a given species, either by tandem or segmental duplications. Annotation transfert between ultraparalogs is more efficient than between orthologous sequences. It is also relevant for functional analysis as ultraparalogs likely share a similar function and are often involved in functional redundancy and/or neofunctionnalization.

### Ortholog prediction by similarity methods

#### Reciprocal Best Hit (RBH) or Best Blast Mutual Hit (BBMH)

This blast-based method is currently the one most frequently used to find one-to-one relationships. It assumes that a reciprocal best hit association between two species proteins defines an ortholog pair. We downloaded 1/1 relationships obtained by BBMH from the OryGenesDB database (E-value cutoff of 0.1) [59,62].

#### Inparanoid

Inparanoid is based on pairwise similarity values calculated in four ways: A/B, B/A, A/A, B/B. Orthologous sequences were detected according to both homology and length cut-off. Orthologs were clustered around the main orthologs for each species (in-paralogs). A confidence value based on bootstrapping technique was assigned for each in-paralog relationship and for each ortholog group itself. We ran Inparanoid [63] on *Oryza sativa *and *Arabidopsis thaliana *whole proteomes (91730 sequences). We only retained ortholog predictions (in-paralogs and out-paralogs) with a bootstrap threshold above 50%.

### Ortholog counting procedure

In order to compare the different prediction methods GreenPhyl, Inparanoid and BBMH, we assumed that predictions are identical between two or three methods when several splice forms of the same locus are predicted as orthologs. For BBMH, all the ortholog predictions are 1/1 relations, but for Inparanoid and GreenPhyl most of them are n1 sequences of *A. thaliana *orthologs to n2 rice sequences. The counting score of an orthologous relationship is computed as n1*n2.

#### Hardware

GreenPhyl runs on an AIX system at the National Computer Center of Higher Education [64] in a multi-job manner using IBM P1600 Power4 node clusters of 16.

## List of abbreviations used

BBMH: Best Blast Mutual Hit; RBH: Reciprocal Best Hit; TF: Transcription factors.

## Authors' contributions

MGC, SG, GD and CP carried out the clustering, GreenPhyl construction and optimization. MGC and CP performed the entire test on GreenPhyl, drafted the paper and conducted all analyses.

## Supplementary Material

Additional File 1 Comparison of DATF, DRTF and semi-automatic clustering for TF families. Click here for file

Additional File 2 List of species name abbreviations recognized by the GreenPhyl pipeline. Click here for file

Additional File 3 Updated NCBI tree of life including the top 100 plant species. Click here for file

Additional File 4 Number of orthologs prediction for TF families by GreenPhyl compared to BBMH and Inparanoid methods. Click here for file

Additional File 5 Extended and In-specific ortholog prediction of GreenPhyl and Inparanoid. Click here for file

Additional File 6 Segmental and tandem duplications detected as Ultraparalogs (UP) and In-paralogs by GreenPhyl and Inparanoid respectively. Click here for file
