# Supplementary material for: Phylogenomics of plant genomes: a methodology for genome-wide searches for orthologs in plants
Source: BMC Genomics. 2008 Apr 21;9:183. doi: 10.1186/1471-2164-9-183 (PMC2377279; doi:10.1186/1471-2164-9-183)
Supplement: Additional File 3 — Updated NCBI tree of life including the top 100 plant species. [file 1471-2164-9-183-S3.doc]

[References: Barns,S.M., and N.R.Page 1996 PNAS 93 9188-9193;

NCBI taxonomy;

"Tree of Life": http://phylogeny.arizona.edu/tree/phylogeny.html;

"Deep Green": http://ucjeps.berkeley.edu/bryolab/greenplantpage.html]

([begin of Viridiplantae Green plants]

([begin of Streptophyta]

(

([begin of Streptophytina]

([begin of Embryophyta]

((

([begin of Tracheophyta]

(

([begin of Spermatophyta]

(((

([begin of Liliopsida]

(

([begin of Commelinids]

(

([begin of Poales]

([begin of PACCADclade]

([begin of Panicoideae]

(([&&NHX:S=SACOF-Saccharum_officinarum:T=4547],[&&NHX:S=SORBI-Sorghum_bicolor:T=4558])[],[&&NHX:S=MAIZE-Zea_mays:T=4577])[&&NHX:S=Andropogoneae:T=147429],

([&&NHX:S=PENGL-Pennisetum_glaucum:T=4541],[&&NHX:S=SETIT-Setaria_italica:T=4555])[&&NHX:S=Paniceae:T=147428]

)[&&NHX:S=Panicoideae:T=147369],

[&&NHX:S=AELLI-Aeluropus_littoralis:T=110874]

)[&&NHX:S=PACCADclade:T=147370],

([begin of BEPclade]

((([&&NHX:S=HORVU-Hordeum_vulgare:T=4513],[&&NHX:S=TRIAE-Triticum_aestivum:T=4565])[],[&&NHX:S=SECCE-Secale_cereale:T=4550])[&&NHX:S=Triticeae:T=147389],

([&&NHX:S=AVESA-Avena_sativa:T=4498],[&&NHX:S=AGRST-Agrostis_stolonifera:T=63632])[&&NHX:S=Aveneae:T=191503]

)[&&NHX:S=Pooideae:T=147368],

[&&NHX:S=ORYZA-Oryza_sativa:T=4530]

)[&&NHX:S=BEPclade:T=359160]

)[&&NHX:S=Poales:T=38820],

([&&NHX:S=MUSAC-Musa_acuminata:T=4641],[&&NHX:S=ORCHID-Orchidantha_sp:T=191989])[&&NHX:S=Zingiberales:T=4618]

)[],

[&&NHX:S=M-Cocos_nucifera:T=13894]

)[&&NHX:S=Commelinids:T=4734],

(

([&&NHX:S=ALLCE-Allium_cepa:T=4679],[&&NHX:S=ASPOF-Asparagus_officinalis:T=4686])[],[&&NHX:S=PHAAO-Phalaenopsis_aphrodite:T=212056]

)[&&NHX:S=Asparagales:T=73496]

)[]

,[&&NHX:S=ACOCL-Acorus_calamus:T=4465]

)[&&NHX:S=Liliopsida:T=4447],

([begin of eudicotyledons]

((

(

((

(

(

(([&&NHX:S=SOLLC-Solanum_lycopersicum:T=4081],[&&NHX:S=SOLBU-Solanum_bulbocastanum:T=147425])[&&NHX:S=Solanum:T=4107]

,[&&NHX:S=ATRBE-Atropa_belladonna:T=33113])[],[&&NHX:S=CAPAN-Capsicum_annuum:T=4072]

)[&&NHX:S=Solanoideae:T=424551],

([&&NHX:S=TOBAC-Nicotiana_tabacum:T=4097],[&&NHX:S=NICPL-Curled_leaved_tobacco:T=4092])[&&NHX:S=Nicotiana:T=4085]

),[&&NHX:S=PETHY-Petunia_hybrida:T=4101]

)[&&NHX:S=Solanaceae:T=4070],

[&&NHX:S=IPOBA-Ipomoea_batatas:T=4120]

)[&&NHX:S=Solanales:T=4069],

([&&NHX:S=ANTMA-Antirrhinum_majus:T=4150],[&&NHX:S=JASNU-Jasminum_nudiflorum:T=126431])[&&NHX:S=Lamiales:T=4143]

)[],

[&&NHX:S=COFAR-Coffea_arabica:T=13443]

)[&&NHX:S=lamiids:T=91888],

(

(

([&&NHX:S=DAUCA-Daucus_carota:T=4039],[&&NHX:S=PETCR-Petroselinum_crispum:T=4043])[&&NHX:S=Apiaceae:T=4037]

,[&&NHX:S=PANGI-Panax_ginseng:T=4054]

)[&&NHX:S=Apiales:T=4036],

([&&NHX:S=HELAN-Helianthus_annuus:T=4113],[&&NHX:S=LACSA-Lactuca_sativa:T=4236])[&&NHX:S=Asterales:T=4209]

)[&&NHX:S=Campanulids:T=91882]

)[&&NHX:S=Asterids:T=71274],

(

(

([begin Rodids]

((

([begin eurosid I]

(

(

([begin of Fabales]

(

(

([begin of Hologalegina]

(((([&&NHX:S=LENCUL-Lens_culinaris:T=3864],[&&NHX:S=VICFA-Vicia_faba:T=3906])[],[&&NHX:S=PEA-Pisum_sativum:T=3888])[],[&&NHX:S=MEDSA-Medicago_sativa:T=3879])[] ,[&&NHX:S=VICFA-Vicia_faba:T=3906])[&&NHX:S=Vicieae:T=163743]

,[&&NHX:S=LOTJA-Lotus_japonicus:T=34305]

)[&&NHX:S=Hologalegina],

(([&&NHX:S=VIGUN-Vigna_unguiculata:T=3920],[&&NHX:S=SOYBN-Glycine_max:T=3847])[],[&&NHX:S=PHAVU-Phaseolus_vulgaris:T=3885] )[&&NHX:S=Phaseoleae:T=163735]

)[],

[&&NHX:S=CICAR-Cicer_arietinum:T=3827]

)[&&NHX:S=Fabaceae:T=3803],

([&&NHX:S=MULAC-Muraltia_acerosa:T=292053],[&&NHX:S=POLVU-Polygala_vulgaris:T=174553])[&&NHX:S=Polygalaceae:T=4274]

)[&&NHX:S=Fabales:T=72025],

([&&NHX:S=CUCSA-Cucumis_sativus:T=3659],[&&NHX:S=CUCME-Cucumis_melon:T=3656])[&&NHX:S=Cucurbitales:T=71239]

),((([&&NHX:S=HEVBR-Hevea_brasiliensis:T=3981],[&&NHX:S=MANES-Manihot_esculenta:T=3983])[&&NHX:S=Crotonoideae:T=235631],[&&NHX:S=RICCO-Ricinus_communis:T=3988])[&&NHX:S=Euphorbiaceae:T=3646],[&&NHX:S=POPAL-Populus_alba:T=43335])[&&NHX:S=Malpighiales:T=3646]

),(([&&NHX:S=MALDO-Malus_domestica:T=3750],[&&NHX:S=FICCA-Ficus_carica:T=3494])[],[&&NHX:S=MORIN-Morus_indica:T=248361])[&&NHX:S=Rosales:T=3744]

)[&&NHX:S=eurosids I:T=91835],

([begin eurosid II]

(

(([&&NHX:S=BRANA-Brassica_napus:T=3708],[&&NHX:S=BRAOL-Brassica_oleracea:T=3712])[&&NHX:S=Brassica:T=3705]

,[&&NHX:S=SINAL-Sinapsis_alba:T=3728])[]

,[&&NHX:S=ARATH-Arabidopsis_thaliana:T=3702]

)[&&NHX:S=Brassicaceae:T=3700],

(

([&&NHX:S=CITSI-Citrus_sinensis:T=2711],[&&NHX:S=ACERU-Acer_rubrum:T=45314])[&&NHX:S=Sapindales:T=41937],

([&&NHX:S=GOSHI-Gossypium_hirsutum:T=3635],[&&NHX:S=CACAO-Theobroma_cacao:T=3641])[&&NHX:S=Malvales:T=41938]

)

)[&&NHX:S=eurosids II:T=91836]

)[],

([&&NHX:S=OENHO-Oenothera_hookeri:T=85636],[&&NHX:S=EUCGG-Eucalyptus_globulus:T=34317])[&&NHX:S=Myrtales:T=41944]

)[]

,[&&NHX:S=PELHO-Pelargonium_hortorum:T=4031]

)[&&NHX:S=Rosids:T=71275]

,[&&NHX:S=VITVI-Vitis_vinifera:T=29760]

)[],

(

(

([&&NHX:S=BETVU-Beat_vulgaris:T=3555],[&&NHX:S=SPIOL-Spinacia_oleracea:T=3562])[&&NHX:S=Amaranthaceae:T=3563]

,[&&NHX:S=DIACA-Dianthus_caryophyllus:T=3570])[],

[&&NHX:S=MESCR-Mesembryanthemum_crystallinum:T=3544]

)[&&NHX:S=Caryophyllales:T=3524]

)

)[&&NHX:S=eudicotyledons:T=71240]

)[],

(([&&NHX:S=LIRTU-Liriodendron_tulipifera:T=3415],[&&NHX:S=CALFE-Calycanthus_floridus:T=212734])[],([&&NHX:S=PIPCE-Piper_cenocladum:T=398741],[&&NHX:S=DRIGR-Drimys_granadensis:T=224735])[])[&&NHX:S=Magnoliids:T=232347]

)[],

([&&NHX:S=NYMAL-Nymphaea_alba:T=34301],[&&NHX:S=AMBTC-Amborella_trichopoda:T=13333])[&&NHX:S=Basal-Magnoliophyta:T=232365]

)[&&NHX:S=Magnoliophyta:T=3398]

,([&&NHX:S=PINTH-Pinus_thunbergii:T=3350],[&&NHX:S=PINKO-Pinus_koraiensis:T=88728])[&&NHX:S=Coniferophyta:T=3312]

)[&&NHX:S=Spermatophyta:T=58024]

,[&&NHX:S=ADICA-Adiantum_capillus_veneris:T=13818]

)[&&NHX:S=Euphyllophyta:T=78536],

[&&NHX:S=HUPLU-Huperzia_lucidula:T=37429]

)[&&NHX:S=Tracheophyta:T=58023],

([begin of Bryophyta]

[&&NHX:S=PHYPA-Physcomitrella_patens:T=3217],[&&NHX:S=TAKCE-Takakia_ceratophylla:T=253518]

)[&&NHX:S=Bryophyta:T=3208]

),[&&NHX:S=MARPO-Marchantia_polymorpha:T=3197])[],

[&&NHX:S=ANTFO-Anthoceros_formosae:T=48387]

)[&&NHX:S=Embryophyta:T=3197]

,[&&NHX:S=CHAGL-Chaetosphaeridium_globosum:T=96477]

)[&&NHX:S=Streptophytina:T=131221],

([&&NHX:S=ZYGCR-Zygnema_circumcarinatum:T=35869],[&&NHX:S=STAPU-Staurastrum_punctulatum:T=102822])[&&NHX:S=Zygnemophyceae:T=131209]

)[],

[&&NHX:S=MESVI-Mesostigma_viride:T=41882]

)[&&NHX:S=Streptophyta:T=35493],

(

([&&NHX:S=OSTTA-Ostreococcus_tauri:T=70447],[&&NHX:S=NEPOL-Nephroselmis_olivacea:T=31312])[&&NHX:S=Prasinophyceae:T=3152],

((

(([&&NHX:S=VOLCA-Volvox_carteri:T=3067],[&&NHX:S=CHLRE-Chlamydomonas_reinhardtii:T=3052])[&&NHX:S=Chlamydomonadales:T=3042],

[&&NHX:S=SCEOB-Scenedesmus_obliquus:T=3088])[&&NHX:S=Chlorophyceae:T=3166],

([&&NHX:S=CHLVU-Chlorella_vulgaris:T=3077],[&&NHX:S=PROWI-Prototheca_wickerhamii:T=3111])[&&NHX:S=Trebouxiophyceae:T=75966]

)[],

[&&NHX:S=PSEAK-Pseudendoclonium_akinetum:T=160070])

)[&&NHX:S=Chlorophyta:T=3041]

)[&&NHX:S=Viridiplantae:T=33090]

[&&NHX:S=CHLVU-Chlorella_vulgaris:T=3077]
